# Evaluation of therapeutic effects of Crocin in patients with polycystic ovary syndrome: A randomized Double-Blind clinical trial

**DOI:** 10.1186/s40780-025-00432-7

**Published:** 2025-08-08

**Authors:** Fatemeh Saghafi, Atiyeh Javaheri, Farhad Mohammadi, Nooshin Hatamizadeh, Fatemeh Khanizadeh, Mahdieh Safakish, Adeleh Sahebnasagh

**Affiliations:** 1 Department of Clinical Pharmacy, Faculty of Pharmacy and Pharmaceutical Sciences Research Center, Shahid Sadoughi University of Medical Sciences, Yazd, Iran; 2 Department of Obstetrics and Genecology, Faculty of Medicine, Shahid Sadoughi University of Medical Sciences, Yazd, Iran; 3 Department of Pharmaceutics, School of Pharmacy, Shahid Sadoughi University of Medical Sciences and health services, Yazd, Iran; 4 Department of Obstetrics and Genecology, Tehran University of Medical Sciences, Tehran, Iran; 5 Department of Medicinal Chemistry, School of Pharmacy, Shahid Sadoughi University of Medical Sciences and health services, Yazd, Iran; 6 Clinical Research Center, School of Medicine, Department of Internal Medicine, Faculty of Medicine, North Khorasan University of Medical Sciences, Imam Ali Hospital, Shahriar Street, North Khorasan Province, Bojnurd, Iran

**Keywords:** Polycystic ovary syndrome, Metformin, Crocin, Acne

## Abstract

**Background:**

Polycystic ovary syndrome (PCOS) is the most common endocrine-metabolic disorder in women of reproductive age. Crocin is known as the main component of saffron, which has antioxidant properties and has the ability to scavenge free radicals. Considering the pathophysiology of PCOS and also the anti-inflammatory and antioxidant properties of crocin in improving insulin resistance, we aimed to evaluate the efficacy of the combination of crocin and metformin compared to metformin alone in PCOS patients.

**Methods:**

This study is a prospective, double-blind randomized controlled clinical trial. Fifty patients with PCOS and hirsutism were included from Baqaipour Obstetrics and Gynecology Clinic in Yazd and randomly assigned into the two study arms of control group [metformin and placebo (*n* = 25)] or intervention group [metformin and crocin (*n* = 25)]. Patients were administered crocin 15 mg tablets once a day in combination with metformin 500 mg twice a day for 12 weeks to complete three full periods of the monthly cycle. The studied endpoints were evaluated at the beginning and end of the study.

**Results:**

Crocin significantly improved the serum level of follicle-stimulating hormone (FSH) (*P* = 0.048) and Ferriman- Gallwey score (*P* = 0.042) at day 90. Furthermore, crocin group had a significant improvement in terms of acne severity at the end of the study (*P* = 0.03), so that none of the patients in the crocin group had severe acne at the end of the study. However, the comparisons between two groups were not statistically significant for other evaluated outcomes including fasting blood sugar (FBS), dihydroepiandrosterone-sulfate (DHEA-S), luteinizing hormone (LH), dermatology life quality index (DLQI), and blood pressure (BP) at the end of the study.

**Conclusion:**

In the present study, concurrent use of crocin along with metformin was significantly effective in ameliorating the unpleasant side effects of PCOS, including hirsutism and acne, and increasing FSH sex hormone levels in patients with PCOS.

**Trial registry date:**

26/08/2021, **Trial Registry number**: IRCT20210730052027N1.

## Background

Polycystic ovary syndrome (PCOS) is a condition characterized by endocrine disorder, anovulation, increasing serum luteinizing hormone (LH) levels and insulin-induced hyperandrogenism. The clinical manifestations of the disease include menstrual disorder, dyslipidemia, acne and hirsutism. The prevalence of PCOS in premenopausal women is approximately 6–21% at reproductive age [1, 2]. PCOS is considered a common treatable causes of female infertility, because of the hormonal imbalance which interferes ovulation [3]. Even in case of fertility, these women will experience a high-risk pregnancy with the possibility of hypertension, pre-eclampsia, and gestational diabetes. Furthermore, PCOS women are at a higher risk of endometrial cancer, cardiovascular, metabolic, and psychological problems which necessitates careful periodic examinations [4].

Among hormonal disturbances, hyperandrogenemia is a key criterion for diagnosis of PCOS. Elevated LH and decreased follicle-stimulating hormone (FSH) levels, in response to androgen excessive secretion, play a major role in clinical manifestations of PCOS [5]. Notably, insulin resistance and hyperinsulinemia have a key role in its underlying pathogenesis [6]. Insulin resistance and hyperinsulinemia further elevates androgen secretion and circulating free testosterone, better known as hyperandrogenism [7]. Studies have shown that PCOS females are usually insulin resistance, which subsequently increase the risk of type 2 diabetes and overweight [8].

Metformin is essential in PCOS treatment by relieving insulin resistance. Metformin enhances insulin sensitivity and helps restore the normal menstrual cyclicity and ovulation [9]. Metformin also acts as an ovulation induction compound in PCOS females. Furthermore, this medication helps with weight loss and, consequently, improves insulin resistance. However, various studies reported the bothersome gastrointestinal side effects of this medication such as flatulence, vomiting and nausea [10].

Considering the limited side effects and high safety profile, medicinal plants and phytoproducts have been proposed as viable alternatives to chemical substances for decades, and their use is increasing worldwide [11]. Crocin, is a bioactive natural product and the main active component of Crocus sativus Linne (saffron). Saffron was mentioned a therapeutic option in gynecological diseases such as primary dysmenorrhea and premenstrual tension syndrome [12]. Various studies have shown the anti-inflammatory, anti-depressant, anti-apoptotic properties, anti-lipid peroxidation and anti-atherosclerosis effects of crocin [13].

Crocin exerts its beneficial effects through down-regulation of crucial proinflammatory enzymes. Among the most important of these enzymes are myeloperoxidase (MPO), cyclooxygenase-2 (COX-2), inducible nitric oxide synthase (iNOS), phospholipase A2, and prostanoids [14]. Notably, the release of reactive oxygen species from proinflammatory molecules causes an inflammatory response. Crocin exerts its anti-inflammatory effects by scavenging free radicals. In some studies, it has been suggested that crocin reduces oxidative stress through the inhibition of NF-κB, iNOS, COX-2 and the expression of TNFα, and as a result, the antioxidant properties of this compound are obtained [15]. Furthermore, crocin helps alleviate the complications of inflammatory diseases such as rheumatoid arthritis, Alzheimer’s disease and cancer [16–18]. Animal studies demonstrated that crocin improves insulin sensitivity and plasma glycemic profile, stimulate insulin secretion, and enhances insulin resistance [19–22]. Administration of crocin significantly improved ovarian cysts and hormonal disorders by reducing the levels of LH, testosterone, β-estradiol and cystic follicles and a remarkable increase in FSH in comparison to non-treated PCOS group in an animal [23]. Clinical studies also reported the beneficial effects of crocin on improving glycemic control. In a randomized clinical trial, supplementation with crocin significantly improved insulin resistance, glycemic control [fasting blood sugar (FBS), hemoglobin A1C], and diminished insulin plasma levels in type-2 diabetes patients [24]. Furthermore, crocin has the potential anti-obesity benefits [25]. In an animal study, treatment with crocin leaded to the return of serum testosterone, FSH, LH, glucose, and insulin levels to normal in comparison to the control group [26]. It appears that saffron extract with its antioxidant and anti-inflammatory properties can be effective on parameters related to PCOS, as a recent study has shown that saffron petal extract was able to significantly decrease the serum levels or inflammatory markers and increase the antioxidant capacity which were impaired in PCOS mice [27].

Considering the underlying pathophysiology of PCOS and the beneficial anti-inflammatory and antioxidant properties of crocin in improving insulin resistance, we hypothesized that crocin may help female PCOS patients. Hence, in the present clinical trial, we aimed to evaluate the efficacy of the supplementation with crocin in PCOS patients.

## Materials and methods

### Study design

Recruitment of eligible women was undertaken at Baghayipoor clinic, which is a university‑affiliated clinic in the center of Iran. This trial was performed in accordance with the Declaration of Helsinki and Good Clinical Practice Guidelines. Study protocol was approved by the local Ethics Committee of Shahid Sadoughi University of Medical Sciences (Ethics ID: IR.SSU.MEDICINE.REC.1399.220). This trial was registered in the Iranian Registry of Clinical Trials (https://irct.ir) with registration number of IRCT20210730052027N1 (26/08/2021). Following a health-screening questionnaire, all volunteers provided a written informed consent. Before the intervention, the study protocol, benefits, and possible side effects were described for patients. Understandable written informed consent was obtained for all patients prior to participation in the study.

### Participants

Inclusion criteria were women aged 18 to 44 years with confirmed PCOS who were referred to the Baghayipoor clinic of Yazd city from June 2021 to July 2022. PCOS was confirmed by a gynecologist according to the modified Rotterdam criteria: presence of two of three of the following criteria: oligo-anovulation, hyperandrogenism, and polycystic ovaries [8]. Exclusion criteria were as follows: Serious medical conditions that prevent the patient from regularly attending follow-up visits such as heart failure, consuming exogenous estrogen and progesterone for menstrual regulation, taking anti-depressant drugs, pregnancy, lactation and unwillingness to continue the study.

### Randomization and blinding

In the current double-blind study (investigators and participants), 50 eligible subjects were randomly assigned into two groups using the random allocation software (version 1) [28]. The generated permutations included ten blocks of five. To run this software output, the qualified patients received number 1 to 50. All steps were covered from the patient, physician, and the investigator who recorded the clinical responses.

### Interventions

Subjects in the intervention group consumed 500 mg metformin twice a day [29] together with 15 mg/day crocin while participants in the control group consumed metformin along with placebo for 12 weeks [30]. Crocin supplementation used in this trial was an extract of Crocus sativus Linne (saffron), manufacturing by Pooyesh daroo sina Com. (Krocina^®^, Mashhad, Iran). Placebo tablets were also purchased from Pooyesh daroo sina Com. The hardness, thickness, color, and odor of placebo tablets were adjusted similar to crocin tablets.

### Outcomes

Demographic data including age, marital status, body mass index (BMI), smoking, and educational status were recorded at baseline. Peripheral blood samples were obtained after an overnight fast to assess clinical laboratory tests at the beginning and the end of the study. The main laboratory parameters were dihydroepiandrosterone (DHEA), FSH, LH as well as FBS. Clinical measurements including scoring of hirsutism, acne, blood pressure (BP), and quality of life were performed at baseline and the end of the study. DHEA level was determined using DHEA sulfate (DHEAS) test [31] and radio-immunoassay (RIA) test was used to determine the levels of LH and FSH [32].

Hirsutism was graded based on the Ferriman-Gallwey scoring system. According to this system, nine anatomical regions (upper lip, chin, chest, upper abdomen, lower abdomen, upper back, lower back, arm, and thigh) were examined and scored between 0 (no terminal hair) and 4 (maximum hair increase) for each region. A total score of 8 or above is considered hirsutism [33].

Dermatology Life Quality Index (DLQI) is a questionnaire consisting of ten items developed specifically for dermatologic diseases. DLQI represents a self-reported rating of hirsutism with a score of 0–30. The average DLQI score is between 0 and 5 in normal population and the maximum score is 30, considered the worst quality of life [34].

### Sample size

The sample size was calculated as 23 per group based on earlier experience [35], and LH standard deviation of 2.15 in order to reach a clinical mean difference of 1.8 and standard effect size of 0.84 using the sample size eq.  $N = \frac{{{{({Z_{\frac{\alpha }{2}}} + {Z_\beta })}^2} \times 2{S^2}}}{{({{\mathop X\limits^ - }_1} - {{\mathop X\limits^ - }_2})}}$, for comparing two means; the estimated sample size was increased to 25 per group to take account of potential attrition 10% (α = 0.05; 1-β = 0.8). The power analysis was performed and the study power based on the LH outcome was 0.55.

### Statistical analysis

The normality of the data was assessed using Kolmogorov-Smirnov tests. All data were processed by statistical package for social science (SPSS) software version 18. The quantitative and qualitative variables were reported as mean (SD), median (IQR), and number (%). Comparisons among quantitative variables were analyzed by independent-samples *t*-test, and comparison among qualitative variables were analyzed by Chi-square. Paired-samples *t*-test and Wilcoxon Signed Ranks Test were performed for comparisons of variables with normal and skewed distribution of data before and after the intervention in each group. All the statistical analysis was conducted by Statistical package for social science (SPSS) software version 23 and *P* < 0.05 was considered as statistically significant.

## Results

**Fig. 1:**
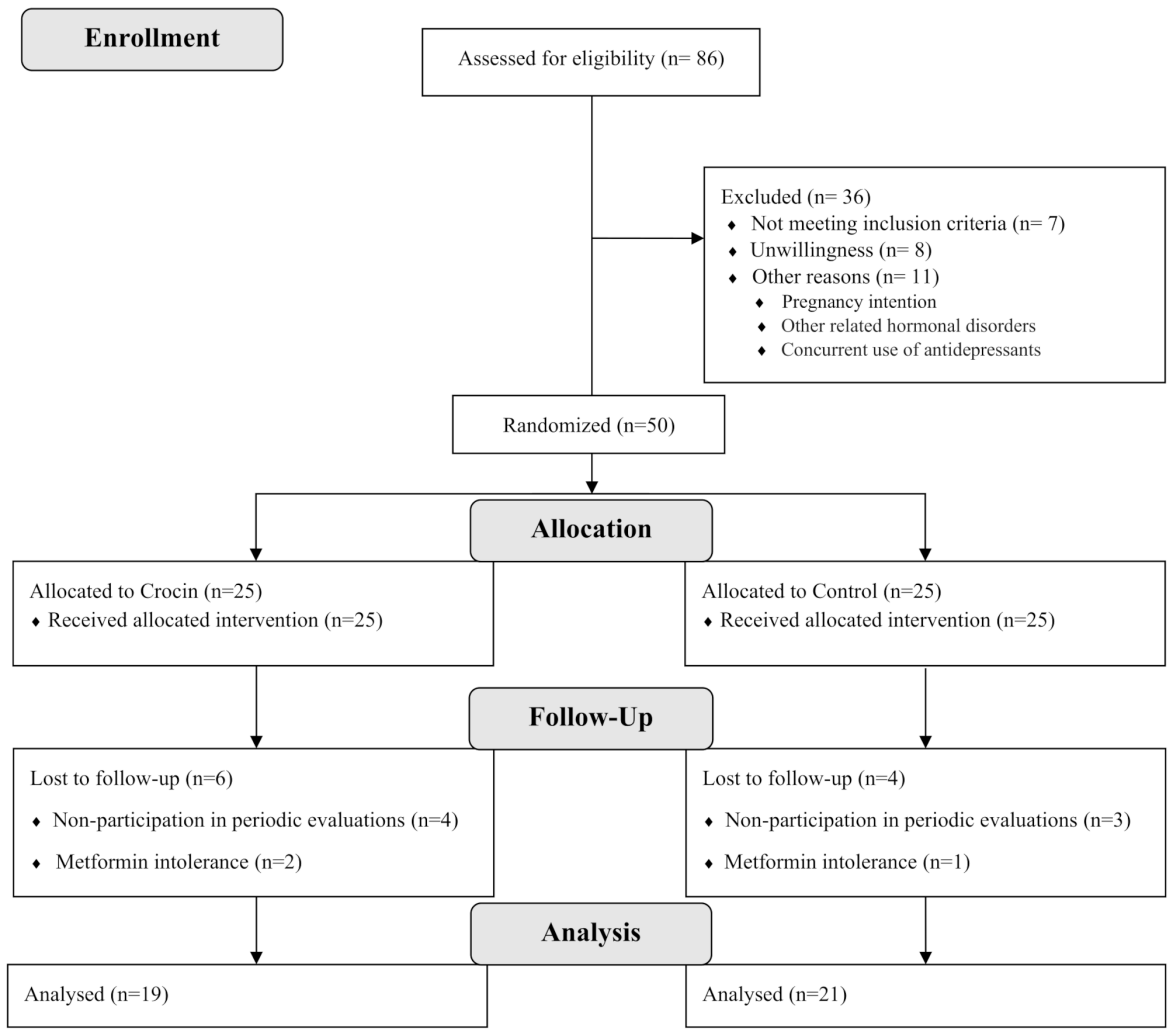
Flow diagram of participant’s recruitment and follow-up

From June 2021 to July 2022, a total of 86 patients with confirmed PCOS were assessed for eligibility; of them, 36 were ineligible for various reasons. During the study follow-up, 6 patients in the control group and 4 patients in the intervention group were excluded from the study because of irregular participation in periodical follow-up visits and metformin intolerance. Eventually, 50 patients were randomized to receive crocin or placebo in combination with metformin (25 per group) (Fig. 1).

The baseline characteristics of the participants were shown in Table 1 and were balanced between groups. The mean (SD) age of participants was 26.35 (4.44) years old and the mean (SD) BMI was 24.58 (4.79). Oligomenorrhea was the most common menstrual disorder at the beginning of the study.

As part of the safety evaluation, the patients were questioned about experiencing possible side effects. Although, some patients complained about sleep disorders, nausea and vomiting, the side effects were so mild that none of the patients discontinued their treatment because of the adverse effects.

**Table 1:** Comparison of demographic variables, menstrual disorder, acne, quality of life between two study groups with PCOS

Parameters	Crocin Group *N* = 19	Placebo Group *N* = 21	*P*
Age (y), *mean (SD)*	25.23 (3.89)	27.57 (4.78)	0.101
BMI (kg/m^2^), *mean (SD)*	24.13 (5.25)	25.08 (4.31)	0.531
Marital status, *F (%)*			
Married	17 (81%)	14 (73/7%)	0.582
Smoking, *F (%)*			
Yes	1 (4.8%)	2 (10.5%)	0.480
Educational status, *F (%)*			0.964
Primary/Secondary school	1 (4.8%)	2 (10.5%)	
Diploma	2 (9.5%)	6 (31.6%)	
Associated Degree	6 (28.6%)	1 (5.3%)	
Bachelor’s degree	10 (47.6%)	8 (42.1%)	
Master’s degree	2 (9.5%)	2 (10.5%)	

BMI: Body mass index; y: year; kg/m^2^: Kilogram per Square Meter; F: Frequency; SD: Standard Deviation; Independent *t*-test and Chi-square were used to compare quantitative and qualitative variables, respectively. *P* < 0.05 is considered as statistically significant

Hirsutism was graded based on the Ferriman-Gallwey scoring system.

### Study outcomes

The results of the present study indicated that both secondary outcomes, FSH (*P* = 0.048) and hirsutism based on Ferriman-Gallwey (*P* = 0.042), were significantly improved in patients supplemented with crocin compared to placebo over the study period while the primary outcome, LH (*P* = 0.201) was insignificant. The comparisons between the two interventions were not statistically significant for FBS, DHEA-S, LH, DLQI, and BP at the end of the study (Table 2).

FSH level was increased significantly only in the crocin group (*P* < 0.001). Furthermore, LH, DLQI, and Ferriman- Gallwey changes in the crocin group were significantly lower after 3-month treatment (*P*-value < 0.001), but FBS, BP, DHEA-S remained unchanged. The evaluation of the Ferriman- Gallwey scores changes in each of the crocin or placebo groups showed a significant reduction in both within and between groups changes (*P*-value < 0.001). In the placebo group, LH levels (*P*-value = 0.016) and Ferriman- Gallwey were decreased significantly at day 90 (*P*-value = 0.015) (Table 2). According to the LH results, the power of the study was 0.55.

**Table 2:** The comparison of evaluated in the participated at baseline and at the end of the intervention

Variable	Crocin Group *N* = 19			Within group *P*	Placebo Group *N* = 21			Within group *P*	Between group *P*
	Before	After	After-Before		Before	After	After-Before		
FBS, mg/dl	99.9 (15.1)	95.2 (11.6)	4.7 (17.8)	0.241	104.6 (23.1)	100.3 (16.3)	4.3 (28.1)	0.511	0.954
DHEA-S, ng/mL	1.7 (1.4)	1.5 (1.5)	0.15 (1.6)	0.660	1.9 (1.3)	2.4 (1.7)	0.5 (2.0)	0.270	0.250
LH, mIU/mL	6.8 (2.9)	3.3 (1.6)	3.49 (2.9)	< 0.001*	6.7 (2.5)	4.5 (1.9)	2.2 (3.6)	0.016*	0.201
FSH, mIU/mL	5.6 (1.7)	7.4 (1.9)	1.8 (1.76)	< 0.001*	5.9 (2.7)	6.6 (1.9)	0.7 (1.7)	0.097	0.048*
DLQI	6.3 (4.6)	3.6 (2.7)	2.7 (4.3)	0.010*	8.3 (4.5)	6.6 (2.7)	1.7 (5.6)	0.205	0.540
Ferriman- Gallwey	7.9 (4.7)	4.1 (3.9)	3.8 (4.2)	< 0.001*	7.6 (3.9)	6.9 (3.9)	1.6 (2.5)	0.015*	0.042*
BP	116.1 (16.2)	114.3 (13.0)	1.9 (18.1)	0.633	113.4 (16.1)	113.6 (15.0)	0.2 (22.5)	0.960	0.740

FBS: Fasting Blood Sugar; DHEA-S: Dihydroepiandrosterone-sulfate; LH: Luteinizing Hormone; FSH: Follicle-Stimulating Hormone; DLQI: The Dermatology Life Quality Index; BP: Blood pressure; * *Independent t-test* was used to analyze the variables before and after the study; *Paired t-test* was used to compare the variables in each group before and after the study; data are based on mean (SD); * *P* < 0.05 is considered as statistically significant

At the initiation of the study, about two-third of the patients in both groups experienced oligomenorrhea. It should be noted that two groups were not significantly different in term of menstrual disorders at day 90 (*P* = 0.53). At the end of the study, the majority of the patients had normal menstrual cycle; so that 19 participants (90.5%) in the crocin group and 13 patients (68.4%) in the placebo group had normal menstrual cycles. Regarding the quality of life, the results of statistical analysis showed that the two groups did not differ significantly in terms of quality of life in the end of the study period (*P* = 0.29). Notably, crocin group had a significant improvement in terms of the severity of acne (*P* = 0.03), so that none of the patients in the crocin group complained severe acne in the last follow-up session (Table 3).

**Table 3:** Comparison of menstrual disorder, acne, and quality of life between two study groups with PCOS

Parameters	Crocin Group *N* = 19		Placebo Group *N* = 21		*P*
	Before	After	Before	After	
Menstrual Disorder, *F (%)*					0.285*
Normal	0 (0.0)	19 (90.5)	1 (5.3)	13 (68.4)	
Amenorrhea	3 (14.3)	1 (4.8)	1 (5.3)	1 (5.3)	
Dysmenorrhea	3 (14.3)	1 (4.8)	4 (21.1)	3 (15.8)	
Oligomenorrhea	15 (71.4)	0 (0.0)	13 (68.4)	2 (10.5)	
Acne, *F (%)*					
No	6 (28.6)	11 (52.4)	4 (21.1)	3 (15.8)	0.029*
Very Mild	4 (19.0)	5 (23.8)	1 (5.3)	2 (10.5)	
Mild	1 (4.8)	3 (14.3)	2 (10.5)	7 (36.8)	
Moderate	7 (33.3)	2 (9.5)	10 (52.6)	5 (26.3)	
Sever	3 (14.3)	0 (0.0)	2 (10.5)	2 (10.5)	
Quality of Life, *F (%)*					
No complication	3 (14.3)	11 (52.4)	1 (5.3)	6 (31.6)	0.291**
Complication once a weak	12 (57.1)	7 (33.3)	10 (52.6)	11 (57.9)	
Complication ≥ twice a weak	6 (28.6)	3 (14.3)	8 (42.1)	2 (10.5)	

F: Frequency; *Extended Fisher’s exact test and **Chi-square test were used to compare variables; *P* < 0.05 is considered as statistically significant; ^1^*P*-value at day 90 between groups; *P* < 0.05 is considered as statistically significant

## Discussion

In this placebo-controlled double-blinded clinical trial, the therapeutic effect of crocin in female patient with PCOS was investigated. The results of the present study demonstrated that concurrent use of crocin along with metformin significantly ameliorates the unpleasant side effects of PCOS, including hirsutism and acne.

Hyperandrogenemia and hyperinsulinemia are important risk factors and diagnostic criteria for PCOS. Increased LH levels and decreased FSH levels due to excessive androgen secretion play an important role in clinical presentations of PCOS [3]. The aqueous extract of saffron, containing crocin, can improve glycemic control with its antioxidant capacity in diabetic rats [36]. The results of a RCT in women with PCOS recently shown that the administration of crocin at the higher dose of 15 mg twice a day with the same duration of 12 weeks improves FBS, lipid profile, insulin and cardioprotective and inflammatory markers in patients supplemented with crocin [37]. In the present study, administration of a lower dose of 15 mg daily of crocin could not affect BP and FBS. Notably, in Rahimi’s study, patients in the control group received only placebo. While in the present study, both groups of patients, along with the intervention, received metformin to control the symptoms of the disease, which can understate the effect-size of the difference between the groups. As described, insulin resistance and hyperinsulinemia play a significant role in the pathogenesis of PCOS [6]. Metformin is the most commonly used treatment to improve insulin sensitivity in insulin-resistant conditions such as diabetes, prediabetes, polycystic ovary syndrome, and obesity [38]. Because it was not ethical to deprive patients of their main treatment, metformin was prescribed to both groups of patients in this study [39].

Previously, the antioxidant effects of crocin have been shown [40–42]. Since oxidative stress plays an important role in the pathophysiology of PCOS, compounds with antioxidant properties have a potential role in the management of PCOS [43]. In several studies, the anti-inflammatory and antioxidant properties of crocin have been investigated. It has been shown that crocin has the potential in the prevention and treatment of various diseases [44]. Particularly, in an animal model of PCOS, treatment with saffron extract increased glutathione and glutathione S-transferase serum levels, which are well known as antioxidant factors [27].

Previous studies have shown that injection of crocin restored the serum levels of testosterone, FSH, LH, glucose, insulin, cholesterol, and serum estradiol levels to normal [26]. In the current study, the increased LH and decreased FSH levels were significantly recovered in crocin group. Sadoughi et al. investigated the effects of crocin on the number of ovarian follicles and the amount of sex hormones in a rat model of PCOS induced by letrozole. Their results showed that the serum level of FSH, the number of Preantral, antral and corpus luteum follicles were significantly increased in the PCOS group treated with a concentration of 100 mg/kg of crocin compared to the untreated PCOS group [23]. Furthermore, in another animal model of PCOS in mice, treatment with saffron petal extract decreased LH, estrogen, testosterone, and increased FSH and progesterone serum levels. It was concluded that saffron petal extract improves PCOS presentations by restoring the estrogenic negative feedback in the pituitary-ovarian system through its antioxidant and anti-inflammatory properties [45].

For the first time, this study also investigated the effects of crocin on the bothersome symptoms of PCOS. As indicated, the results of this study showed that crocin significantly ameliorated hirsutism and acne compared to the control group. It should be noted that acne is one of the common dermatological manifestation of the disease, with a prevalence of 43% in PCOS patients [46]. In other words, one of the causes of acne in women is PCOS. As the results of this study indicated, crocin significantly improved acne compared to the control group. It was shown that crocin can improve prostate hyperplasia caused by testosterone reduction in mice [47]. Since the increase of androgens play a key role in causing hirsutism [48], it seems that crocin exerts its beneficial effects by reducing the production of androgens. As indicated, crocin also significantly ameliorated hirsutism.

Although the results observed in this trial were promising, the limitations of the study should be taken into account when interpreting the results. First is the small size of the participants. Although 86 patients were evaluated for eligibility, only a limited number of them completed the study, due to the COVID-19 pandemic. As mentioned, for the first time ever, the bothersome symptoms of PCOS were measured in this study and the hormonal parameters of the patients were examined. However, all variables that could play a role in the pathophysiology of this disease were not evaluated. Furthermore, we did not measure the fertility status of the included patients at the beginning and end of the study which should be addressed in future studies. It is also suggested to prescribe a higher dose of crocin 15 mg twice a day. Perhaps the reason for not observing the positive effects of crocin on endocrine parameters was the administration of a lower dose of crocin in this study. We recommend in addition to the endpoints measured in this study, other important parameters of insulin levels, body weight, inflammatory markers, cardioprotective indices, and lipid profile be evaluated in future studies. Furthermore, the effects of longer duration of administration of this herbal supplement should be assessed to determine the final outcome of PCOS patients. Last but not least, the safety of crocin should be examined. As there are still some concern regarding the hepatotoxic effects of crocin [49] and we suggest periodic measurement of liver function in patients receiving crocin.

## Conclusions

The results of this randomized placebo-controlled clinical trial were suggestive of the concurrent use of crocin along with metformin in patients with PCOS in ameliorating the unpleasant side effects of PCOS, including hirsutism and acne, and increasing FSH sex hormone levels.

## Data Availability

All data generated or analyzed during this study are included in this published article.

## Abbreviations

PCOS: Polycystic ovary syndrome
FSH: Follicle-stimulating hormone
LH: Luteinizing hormone
FBS: Fasting blood sugar
DHEA-S: Dihydroepiandrosterone-sulfate
DLQI: Dermatology life quality index: BP: blood pressure
SD: Standard Deviation
BMI: Body Mass Index
IL-6: Interleukin 6
TNF-α: Tumor necrosis factor-α
